# Development and Application of Children's Sex‐ and Age‐Specific Fat‐Mass and Muscle‐Mass Reference Curves From Dual‐Energy X‐Ray Absorptiometry Data for Predicting Cardiometabolic Risk

**DOI:** 10.1111/ijpo.70051

**Published:** 2025-08-29

**Authors:** Stephanie Tanasia Saputra, Andraea Van Hulst, Mélanie Henderson, Simone Brugiapaglia, Claudia Faustini, Lisa Kakinami

**Affiliations:** ^1^ Department of Mathematics and Statistics Concordia University Quebec Canada; ^2^ Ingram School of Nursing McGill University Quebec Canada; ^3^ Centre de Recherche CHU Sainte‐Justine, Université de Montréal Quebec Canada; ^4^ Department of Pediatrics Université de Montréal Quebec Canada; ^5^ School of Public Health Department of Social and Preventive Medicine, Université de Montréal, Montréal Quebec Canada; ^6^ School of Health, Concordia University Quebec Canada

**Keywords:** adiposity, cardiometabolic risk, dual‐energy x‐ray absorptiometry, reference curves, youth

## Abstract

**Background:**

A dual‐energy x‐ray absorptiometry (DXA)‐derived phenotype classification based on fat mass and muscle mass has been developed for adults. We extended this to a paediatric population.

**Methods:**

Children's (≤ 17 years) DXA data in NHANES (*n* = 6120) were used to generate sex‐ and age‐specific deciles of appendicular skeletal muscle mass index and fat mass index with the Lambda Mu Sigma method. Four phenotypes (high [H] or low [L], adiposity [A] and muscle mass [M]: HA‐HM, HA‐LM, LA‐HM, LA‐LM) were identified based on being above/below the median compared to same‐sex and same‐age peers. These reference curves were applied to the QUALITY cohort (*n* = 630, 8–10 years of age in 2005) to assess whether the phenotypes correctly identified cardiometabolic risk at baseline, follow‐up (2008–2010), and their longitudinal changes. Multiple linear regression models were adjusted for age, sex, and Tanner's stage.

**Results:**

Compared to the LA‐HM reference group, the HA‐HM phenotype was associated with less favourable HDL, triglycerides, and HOMA‐IR at baseline and first follow‐up, but not in their changes. The HA‐LM phenotype was associated with less favourable HOMA‐IR at baseline and first follow‐up.

**Conclusions:**

Results suggest that phenotypes based on fat and muscle mass may have clinical utility in children and should be further investigated.

## 1 Introduction

In Canada, an estimated 30% of children have overweight or obesity [1] while in the United States, the prevalence is roughly 35% [2]. Globally, the prevalence of overweight/obesity in childhood has risen from 4% to 18% in the past 40 years [3]. As childhood obesity is associated with a higher likelihood of chronic diseases in adulthood such as stroke, heart disease, and diabetes, it is a global public health concern [1]. Body mass index (BMI) is commonly used as a clinical tool for determining overweight/obesity [4]. However, it does not distinguish between fat and muscle mass, which can lead to obesity misclassification due to over/underestimation of fat mass. For instance, people with sarcopenic obesity (characterised by excessive fat mass in the presence of reduced muscle mass) have higher rates of illness, disability, and mortality compared to people with either low muscle mass or obesity [5] but are oftentimes misclassified by BMI. Thus, traditional measures such as BMI have recently been deemed insufficient for a clinical obesity diagnosis [6]. Accordingly, more precise methods to enhance the assessment of body composition and its association with cardiovascular risk are needed.

For instance, dual‐energy x‐ray absorptiometry (DXA) partitions the body into fat and muscle and can be used to calculate derivative values such as fat mass index (FMI: fat mass/height^2^) and appendicular skeletal muscle index (ASMI: appendicular lean mass/height^2^). These indices are commonly used as they account for height and better explain body composition in relation to the ratio of muscle mass and fat mass [7]. In 2014, Prado et al. [8] created sex‐ and age‐adjusted reference curves for adults using cut‐offs for low (< 50th percentile) or high (≥ 50th percentile) mass values to classify four mutually exclusive phenotypes: low adiposity with high muscle mass (LA‐HM), high adiposity with high muscle mass (HA‐HM), low adiposity with low muscle mass (LA‐LM), and high adiposity with low muscle mass (HA‐LM). Using these phenotypes, HA‐LM was associated with lower insulin sensitivity [9].

However, for youth populations, no adiposity and muscle mass DXA reference curves and phenotypes analogous to Prado's work currently exist. Thus, the objective of this research was to develop reference curves and body composition phenotypes for children based on their FMI and ASMI data analogous to those proposed by Prado et al. (Primary objective) and to determine whether these DXA‐derived muscle‐ and fat‐mass phenotypes in children were cross‐sectionally and longitudinally associated with cardiometabolic risks (Secondary objective).

## 2 Methodology

### 2.1 Primary Objective: Development of the Reference Curves

#### 2.1.1 Study Sample

The National Health and Nutrition Examination Survey (NHANES) is a collection of surveys that are used to evaluate the health and nutritional status of the general US population. Since 1999, cross‐sectional samples have been collected every 2 years [10]. Individuals were invited to participate in NHANES using a complex multistage probability sampling technique, in order to provide a representative sample of the general US population [10]. All participants provided informed consent and ethics approval was obtained from the National Center for Health Statistics Ethics Review Board [11] in accordance with the Declaration of Helsinki.

To be consistent with the years used for Prado's reference curves for adults, the same cross‐sectional years from NHANES were used for this study (1999–2006, *n* = 41 474). Respondents were excluded if they were younger than the minimum age (8 years of age) required for DXA scans (*n* = 8655), older than 18 years (*n* = 22 624), or if they had no DXA data due to height/weight restrictions (weight limit of 136 kg, height limit of 196 cm) or pregnancy (*n* = 4075). The final analytical sample was 6120 (3708 male and 2412 female). NHANES used a multiple imputation method to handle missing DXA data [12]. This was incorporated into the data analysis, as described in a later section.

#### 2.1.2 Anthropometric Measures and Body Composition

All body measures were obtained by trained health technicians in accordance with standard protocols. Whole‐body DXA scans were taken with a Hologic QDR‐4500A fan‐beam densitometer (Hologic Inc., Bedford, Massachusetts; software version of 8.26) [12]. Quality control was maintained throughout the DXA data collection and scan analysis, including a phantom scanning schedule. A detailed description of data acquisition and precision can be found in the official NHANES documentation [13, 14].

Following the literature's guidelines, appendicular skeletal mass index (ASMI, representing muscle mass) was calculated using the DXA lean mass for arms and legs, divided by height squared, and FMI (representing fat mass) was calculated by dividing total fat mass by height squared [8, 15, 16]. These values then informed the development of the reference curves, as described in the subsequent section.

#### 2.1.3 Statistical Analysis

To create ASMI and FMI reference curves for children, the LMS (Lambda Mu Sigma) method [17] was used. The LMS method is a curve fitting procedure used to develop reference curves in children and youth [17] and presents a way of obtaining normalised growth centiles after accounting for the skewness that is often present in the data distribution [18]. The method has been extensively explained in the literature [18] and is only briefly described here. First, the median $\left(\mu \right)$ of the variable of interest is calculated as well as the coefficient of variation, *σ*. Next, the Box‐Cox power transformation is applied to the data, under the parameter λ. After the transformation, *z*‐scores and centiles can be calculated through the standard normal distribution assumption which are then plotted into reference curves.

Similar to Prado et al.'s work, the analysis in this study pooled the five DXA imputations available from NHANES while also incorporating the sample weight for each observation [8]. After verifying that the LMS values and corresponding deciles were consistent with the original Prado et al.'s work in the same sample of adults (using lmsChartMaker Pro version 2.54) [19], the methodology was extended to the NHANES data for participants aged 8 to 17 years.

To identify the best fitting model, goodness‐of‐fit measures for the centile curves such as penalised deviance and AIC, as well as some qualitative measures (detrended Q‐Q plot and Q test) were assessed [7, 20]. The curves' complexity was measured by the equivalent degrees of freedom (edf) to measure the smoothness and fluctuations of a curve. The final edf was chosen based on the smoothness of the centile curves and fluctuations of the model‐fit statistics to detect over−/under‐smoothing. The penalised deviance and AIC were monitored to balance the tradeoff between accuracy and parsimony. Additionally, the detrended Q‐Q plot and Q‐tests were assessed, demonstrating that the Q‐statistics of our final edf's were adequate in terms of normality, though some degree of kurtosis was present. Model fit was also assessed after maximising all edf settings and compared to the final edf settings. Distributions between these models were consistent with one another, strengthening our confidence in the edf settings.

After fitting the curves and selecting the optimal edf values, the sex‐stratified deciles for each age group were obtained using LMS and penalised likelihood methods [21]. Using Prado et al.'s approach to classifying participants (at or above/below the median), the FMI and ASMI were used to define four mutually exclusive body‐composition phenotypes: high adiposity and high muscle (HA‐HM), high adiposity and low muscle (HA‐LM), low adiposity and high muscle (LA‐HM) and low adiposity and low muscle (LA‐LM) [8, 22].

### 2.2 Secondary Objective: Application of the Reference Curves

#### 2.2.1 Study Sample

The Quebec Adipose and Lifestyle InvesTigation in Youth (QUALITY) cohort is an ongoing longitudinal study that aims to investigate the natural history of obesity in children. It used a school‐based sampling strategy; eligible participants consisted of Caucasian children aged 8–10 years with at least one biological parent with obesity at baseline [23]. Informed consent to participate was provided by parents, and participating children provided verbal assent. The QUALITY study was approved by the Ethics Review Boards of the CHU Sainte‐Justine and the Quebec Heart and Lung Institute in accordance with the Declaration of Helsinki; this secondary data analysis was approved by Concordia University's ethics board (#0000840540).

A total of 630 families participated at the baseline visit (V1) which was completed between 2005 and 2008. The first follow‐up (V2), when the youth were 10–12 years old, was conducted between 2007 and 2011 and completed for 89% of the original cohort (564 families). Out of the original cohort, 377 families remained for the second follow‐up (V3), which was conducted between 2012 and 2016, when the youth were approximately 15 to 17 years old. To maximise the sample size, participants on the cusp between youth and adulthood reference curves (18 and 19 year olds in V3) were retained. However, as our curves were only developed based on children aged 8–17, participants who were 18–19 year old at V3 (*n* = 54) were categorised based on Prado et al.'s adult decile values cutoffs [8]. The compatibility between our decile values and Prado et al.'s decile values [8] is described in the results.

#### 2.2.2 Measures

Anthropometric measurements such as height, weight, and waist circumference were taken according to standardised protocols [24]. Body composition was assessed with DXA (Prodigy Bone Densitometer System, DFþ14664, GE Lunar Corporation, Madison, WI, USA). Children's sexual stage of maturity was assessed according to the five Tanner stages at each visit by trained nurses [25, 26]. Due to the majority of participants being pre‐pubertal at baseline, pubertal status was defined as ‘pre‐pubertal’ (Tanner stage 1), or ‘pubertal’ (Tanner stage 2–5). A more detailed description of the study design and methods has been previously published [23, 27].

During each visit, blood was drawn by venipuncture after an overnight fast. Samples were immediately stored on ice and then centrifuged, aliquoted, and stored at −80°C until analysis. All biochemistry analyses were conducted at the Department of Clinical Biochemistry of the CHU Sainte‐Justine, which regularly participates in provincial and international quality control programmes and is accredited by the International Federation of Clinical Chemistry [23].

Fasting cardiometabolic risk measures included: total cholesterol (TC), high‐density lipoprotein cholesterol (HDL), low‐density lipoprotein cholesterol (LDL), triglycerides, and glucose. Homoeostatic model assessment of insulin resistance (HOMA‐IR) was calculated as a derivative variable: (fasting insulin (pmol/L))/7.175 × (fasting glucose (mmol/L))/22.5 [28]. In addition, child's central fat was calculated as a derivative variable: (DXA‐measured truncal fat mass in grams/total fat mass in grams) × 100.

#### 2.2.3 Statistical Analysis

In addition to participants lost to follow‐up (*n* = 66 at V2, *n* = 187 at V3), there were also some missing biochemical analysis data (< 1% at baseline, 9%–15% at first follow‐up, and over 50% at second follow‐up). To avoid biased results due to listwise deletion, multivariate imputation by chained equations (MICE) was used for baseline and first follow‐up [29]. Due to significant data missingness at the second follow‐up, analyses are focused on baseline and first follow‐up; the second follow‐up was assessed in a sensitivity analysis. Additional auxiliary variables for MICE included waist circumference, central fat percentage, the children's self‐report of usual amount of time spent watching TV, video, and/or the computer (hours/day) on a typical weekday and weekend, the parental report of previous year's annual household income, and the highest education obtained by either parent. All statistical analyses were conducted with R (version 4.2.0). Ten imputed datasets were created, with all estimates pooled in accordance with the guidelines on multiple imputation [30].

T‐tests, chi‐square tests, and ANOVA tests were conducted to determine the relationship between phenotype classifications and cardiometabolic risk factors at all visits. Statistical comparisons between complete cases and those lost to follow‐up were also conducted. Lastly, multiple linear regression was performed to observe the cross‐sectional relationship between phenotype classification and each of the cardiometabolic risk factors after adjusting for covariates (age, sex, Tanner stage) measured at the same respective timepoint. The change in cardiometabolic measures (∆first follow‐up‐baseline) for phenotypes at baseline, controlling for sex, age at first follow‐up, pubertal status at first follow‐up, and baseline cardiometabolic measure was also assessed. Due to high data missingness, no regression models for the second follow‐up were conducted.

Other covariates/auxiliary variables previously described (children's screen time, household's previous year's annual income, and the highest education obtained by either parent) were not statistically significant in the regression models. As the results were consistent with or without these additional covariates, they were omitted from the final model to minimise overfitting. Finally, the *p* values were extracted from all models across all timepoints (baseline, first follow‐up, and the change between them), where the Benjamini‐Hochberg method was used to adjust for multiple testing (a total of 54 comparisons) [31].

## 3 Results

### 3.1 Primary Objective: Development of Centile Curves

The final edf settings for FMI and ASMI for females and males are presented (Table 1). There were no significant differences between distributions of the final edf and maximum edf when looking at the phenotype classifications in participants (data not shown). The complete L, M, S, and decile values of our reference curves for children aged 8 to 17 years can be found in Tables S1–S4.

**TABLE 1:** Selecting the final edf settings for female and male reference curves.

edf	Female			
	ASMI		FMI	
	Penalised deviance	AIC	Penalised deviance	AIC
3,5,3	6703.1	6719.8	**11728.6** ^a^	**11745.0** ^a^
4,6,4	6697.0	6720.6	11720.8	11743.4
5,7,5	6691.8	6720.9	11714.9	11742.9
6,8,6	6687.0	6722.9	11709.1	11742.7
7,9,7	6682.9	6724.6	11703.2	11743.3
6,5,3	**6694.1** ^a^	**6715.8** ^a^	N/A	N/A
7,9,3	N/A	N/A	11708.5	11739.8
10,10,10	6674.0	6733.8	11689.3	11747.9

edf	MALE			
	ASMI		FMI	
	Penalised deviance	AIC	Penalised deviance	AIC
3,5,3	10803.8	10808.0	16839.3	16856.2
4,6,4	10779.7	10789.4	**16832.8** ^a^	**16856.1** ^a^
5,7,5	10761.1	10779.8	16828.2	16 858.0
6,8,6	10748.4	10776.7	16824.7	16861.1
7,9,7	10739.3	10776.3	16821.6	16864.9
8,10,8	10731.8	10777.3	N/A	N/A
6,6,8	**10747.1** ^a^	**10772.3** ^a^	N/A	N/A
10,10,10	10721.0	10780.7	16816.1	16874.5

^a^ Denotes final edf settings selected based on lowest deviance and AIC alongside QQ plots.

**FIGURE 1:**
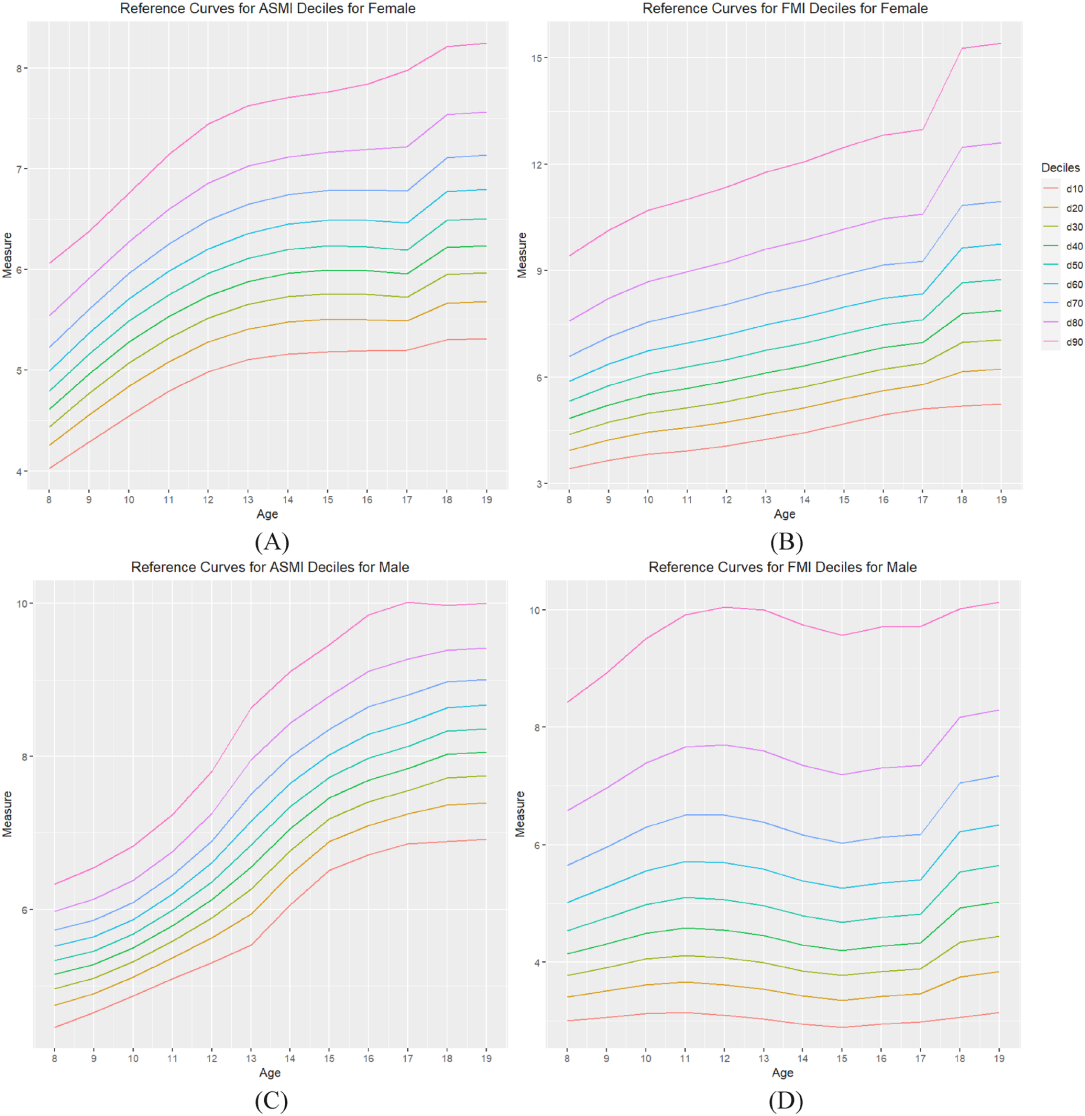
ASMI and FMI reference curves for female (A, B) and male (C, D) age 8–17 years.

For the male and female ASMI curves, the curves were smooth across all ages. However, the FMI curves in males showed a noticeable drop at age 15 years (Figure 1, Panel D). The ASMI curves were steeper in males than in females (Figure 1, Panels A and C), but a noticeable increase occurred from 8 to 13 years old. The FMI curves in females (Figure 1, Panel B) showed a constant upward trajectory from ages 8 to 17, while the FMI curves in males (Figure 1, Panel D) decreased from age 12–15 years.

#### 3.1.1 Compatibility With Prado Et al.'s Curves in Adults

In the transition between our reference curves and Prado et al.'s curves, the 50th percentile at 17 years old to Prado's 50th percentile at 18 years old increases by approximately one unit for females' FMI (7.618 and 8.658, respectively), but the transition for males' FMI and ASMI is smoother (Figure 1).

### 3.2 Secondary Objective: Application of Reference Curves

There were no significant differences in age, screen use, waist circumference, central fat percentage, HDL, triglycerides, glucose, and HOMA‐IR levels in participants who were lost to follow‐up at V2 compared to those who were retained (data not shown). However, participants lost to follow‐up had a lower average household income, with differences also observed in FMI, LDL, and TC. In contrast, there were no statistically significant differences in V2 characteristics between those retained and those lost to follow‐up at V3. As all imputed values were in accordance with van Buuren and Groothuis‐Oudshoorn's guidelines [29], the imputation likely did not severely alter data distributions. Characteristics of the pooled imputed dataset are presented (Table 2). The phenotype classification in QUALITY participants was similar before and after the imputation, where LA‐LM groups were the most frequent phenotype, followed by HA‐HM.

**TABLE 2:** Pooled characteristics of the MICE‐imputed dataset, by sex.

Characteristics	Male $\left(n=343\right)$			Female $\left(n=287\right)$		
	V1	V2	V3	V1	V2	V3
Age (years), mean (SD)	9.62 (0.89)	**11.72 (0.92)**	**16.70 (0.94)**	9.57 (0.95)	**11.57 (0.97)**	**17.00 (1.10)**
Tanner's stage (%)						
Not in pubertal age	**90.67**	**45.19**	2.33	**63.76**	**17.42**	3.14
Parent's education (%)						
1 or both parents with university degree	54.52	48.69	N/A	54.00	51.92	N/A
Household income, mean (SD)	42 713 (18.8 K)	48 781 (21.7 K)	55 416 (24.8 K)	42 099 (18.3 K)	48 122 (22.8 K)	56 354 (24 K)
Screen use (hours/week), mean (SD)	**6.99 (4.34)**	**10.60 (5.23)**	**9.75 (4.63)**	**5.36 (4.17)**	**8.03 (4.16)**	**8.46 (4.64)**
Waist circumference (cm), mean (SD)	67.41 (12.22)	73.07 (13.59)	**82.12 (12.58)**	67.54 (12.05)	71.19 (12.11)	**76.56 (11.84)**
Central fat (%)	**39.91 (5.36)**	**41.72 (4.78)**	**47.59 (5.26)**	**41.68 (5.54)**	**43.41 (4.66)**	**45.77 (4.20)**
ASMI (kg/m^2^), mean (SD)	**5.66 (0.65)**	**6.20 (0.87)**	**7.95 (0.95)**	**5.33 (0.69)**	**5.78 (0.77)**	**6.31 (0.77)**
FMI (kg/m^2^), mean (SD)	**5.01 (3.42)**	**5.94 (3.76)**	**5.55 (3.77)**	**6.10 (3.40)**	**6.84 (3.66)**	**8.57 (4.00)**
Cardiometabolic risks, mean (SD)						
Total cholesterol (mmol/L)	**3.87 (0.68)**	3.76 (0.66)	**3.64 (0.65)**	**3.99 (0.72)**	3.76 (0.67)	**3.84 (0.70)**
HDL (mmol/L)	**1.21 (0.26)**	1.18 (0.26)	**1.08 (0.22)**	**1.16 (0.24)**	1.16 (0.24)	**1.19 (0.24)**
LDL (mmol/L)	**2.31 (0.57)**	2.25 (0.56)	**2.14 (0.53)**	**2.43 (0.60)**	2.23 (0.58)	**2.23 (0.59)**
Triglycerides (mmol/L)	**0.77 (0.37)**	0.74 (0.42)	0.93 (0.51)	**0.89 (0.44)**	0.80 (0.41)	0.91 (0.39)
Glucose (mmol/L)	**4.99 (0.36)**	**5.09 (0.35)**	**5.26 (0.36)**	**4.89 (0.36)**	**5.02 (0.37)**	**4.91 (0.41)**
HOMA‐IR	**0.93 (0.61)**	**1.33 (0.86)**	1.53 (0.99)	**1.18 (0.83)**	**1.64 (1.17)**	1.43 (0.81)
Phenotype (%)						
HA‐HM	26.82	27.70	26.82	32.75	30.31	26.83
HA‐LM	13.70	22.16	17.20	12.54	14.63	16.03
LA‐HM	16.91	11.08	17.49	13.24	11.85	14.63
LA‐LM	42.57	39.06	38.49	41.47	43.21	42.51

*Note*: Boldface indicates the *p* value comparing male and female participants was significant (< 0.05) at that timepoint.

Abbreviations: ASMI: appendicular skeletal muscle index; FMI: fat mass index; HA‐HM: high‐adiposity, high‐muscle; HA‐LM: high‐adiposity, low‐muscle; LA‐HM: low‐adiposity, high‐muscle; LA‐LM: low‐adiposity, low‐muscle.

#### 3.2.1 Regression Results

The results from Table 3 did not differ when stratified by sex; thus, the full sample adjusted for sex is uniformly presented. After adjusting for covariates in the model, compared to the reference group of LA‐HM, both HA‐HM and HA‐LM were significantly associated with less favourable HDL, triglycerides, and HOMA‐IR at baseline and first follow‐up (*p* value < 0.05). Compared to the LA‐HM reference group, participants in the HA‐HM and the HA‐LM phenotype showed more adverse changes in HOMA‐IR in the change between baseline and first follow‐up.

**TABLE 3:** Pooled multiple linear regression results for the association between phenotypes and cardiometabolic measures.

		Total cholesterol^a^, ^b^ B (CI)	HDL^a^, ^b^ B (CI)	LDL^a^, ^b^ B (CI)	Triglycerides^a^, ^b^ B (CI)	Glucose^a^, ^b^ B (CI)	HOMA‐IR^a^, ^b^ B (CI)
V1	HA‐HM	0.15 (−0.03, 0.32)	**−0.16 (−0.22, −0.10)**	**0.17 (0.03, 0.32)** ^c^	**0.30 (0.20, 0.39)**	0.03 (−0.06, 0.12)	**0.76 (0.61, 0.91)**
	HA‐LM	0.11 (−0.09, 0.32)	**−0.07 (−0.15, 0.00)** ^c^	0.13 (−0.04, 0.30)	**0.13 (0.02, 0.24)** ^c^	0.03 (−0.07, 0.14)	**0.31 (0.13, 0.48)**
	LA‐LM	−0.06 (−0.22, 0.10)	0.01 (−0.05, 0.07)	−0.05 (−0.18, 0.09)	−0.05 (−0.14, 0.04)	**−0.09 (−0.18, −0.01)** ^c^	−0.08 (−0.22, 0.06)
	LA‐HM	Reference	Reference	Reference	Reference	Reference	Reference
V2	HA‐HM	0.14 (−0.05, 0.33)	**−0.15 (−0.21, −0.08)**	0.15 (−0.02, 0.32)	**0.31 (0.19, 0.41)**	0.06 (−0.04, 0.16)	**1.06 (0.81, 1.29)**
	HA‐LM	0.10 (−0.10, 0.31)	−0.06 (−0.13, 0.01)	0.10 (−0.09, 0.27)	**0.16 (0.03, 0.28)** ^c^	0.03 (−0.08, 0.14)	**0.45 (0.20, 0.71)**
	LA‐LM	−0.04 (−0.22, 0.15)	0.02 (−0.05, 0.08)	−0.07 (−0.24, 0.11)	0.02 (−0.09, 0.13)	−0.05 (−0.15, 0.05)	−0.14 (−0.32, 0.12)
	LA‐HM	Reference	Reference	Reference	Reference	Reference	Reference
Change (V2‐V1)	HA‐HM	0.07 (−0.14, 0.29)	−0.04 (−0.13, 0.05)	0.09 (−0.06, 0.25)	0.09 (−0.07, 0.26)	0.04 (−0.09, −0.48)	**0.40 (0.13, 0.66)**
	HA‐LM	0.04 (−0.17, 0.25)	−0.02 (−0.10, 0.05)	0.06 (−0.09, 0.22)	0.03 (−0.14, 0.19)	0.06 (−0.08, 0.19)	**0.35 (0.04, 0.66)** ^c^
	LA‐LM	0.04 (0.08, 0.20)	0.04 (−0.02, 0.10)	−0.006 (−0.13, 0.12)	0.003 (−0.14, 0.15)	0.006 (−0.10, 0.11)	−0.07 (−0.33, 0.19)
	LA‐HM	Reference	Reference	Reference	Reference	Reference	Reference

Abbreviations: ASMI: appendicular skeletal muscle index; FMI: fat mass index; HA‐HM: high‐adiposity, high‐muscle; HA‐LM: high‐adiposity, low‐muscle; LA‐HM: low‐adiposity, high‐muscle; LA‐LM: low‐adiposity, low‐muscle.

^a^ Models adjusted for age, sex, and Tanner's stage.

^b^ Boldface indicates the phenotype had a *p* value below 0.05.

^c^ No longer statistically significant after controlling for multiple comparisons.

After adjusting for multiple comparisons using the Benjamini‐Hochberg method [31], compared to the LA‐HM reference group, the association between HA‐LM with HDL at baseline, triglycerides at baseline and first follow‐up, and the change in HOMA‐IR were no longer significant. Moreover, HA‐HM was no longer associated with less favourable LDL at baseline, nor was LA‐LM associated with glucose at baseline.

## 4 Discussion

To the best of our knowledge, this is the first study to develop body‐composition phenotypes based on ASMI and FMI age‐ and sex‐adjusted reference curves with the LMS method for children aged 8 to 17 years old, extending the existing literature [32]. More specifically, we extended Prado's research by (1) creating children's reference curves using a large, representative dataset of the general US population (NHANES) and (2) testing the significance with a cohort of Canadian children [8]. The two high adiposity phenotypes were associated with more adverse HDL, triglycerides, and HOMA‐IR at both timepoints as well as longitudinally, which is consistent with the literature [5, 9, 33]. Although some of these associations were no longer significant after adjusting for multiple comparisons, it is important to interpret results cautiously both before and after the adjustment. While not adjusting increases the risk of Type I errors, applying corrections with the Benjamini‐Hochberg method [31] can raise the risk of Type II errors. In building the reference curves, the identification of appropriate edf for each curve is a critical step. Choosing the edf to smooth a curve has been a subjective exercise or even a ‘black art’ as mentioned by Cole [21]. However, results were similar when comparing multiple edf settings, indicating the misclassification rate is small. The negative impacts of misclassification are further minimised by this study's large sample size.

At baseline, the largest proportion of the QUALITY sample belonged to the LA‐LM phenotype, followed by HA‐HM. This distribution may be a reflection of the QUALITY cohort, which was designed to capture the natural history of obesity. Thus, QUALITY is not a representative Canadian sample, and study results may not generalise to other populations. As the majority of QUALITY participants at baseline were prepubertal, yet most had initiated puberty by the first follow‐up, these later pubertal stages were combined to maximise statistical power. The full spectrum of puberty and how it relates to these phenotypes and cardiometabolic risk should be explored in a larger, longitudinal cohort. Future work should consider adding a representative Canadian population while building the curves so that the results can be more widely applicable to a Canadian population. Relatedly, further research in larger longitudinal samples to capture different moments of the developmental growth period is needed.

As this study focused on cardiometabolic risks that are considered complications of obesity [34], abnormal values might not yet be detectable in a youth sample. Thus, it may be better to assess the phenotypes' significance with other measures that have a more direct significance for children such as fitness assessment and VO2 max (maximal oxygen uptake) [35, 36]. This study used the FMI and ASMI median to be consistent with the methodology proposed by Prado et al. [8] However, the overall weak association of our phenotypes might be due to the fact that the phenotypes were built based on a simple 50th percentile cutoff method. This method might be oversimplified, and further work is needed to classify phenotypes based on clinically meaningful thresholds connected with health outcomes. Relatedly, other methods such as the Box‐Cox power exponential transformation could be used to develop the reference curves [37].

This study created reference curves for 8–17 year olds, and merged these values with Prado et al.'s [8], which were constructed for 18–85 year olds. However, additional methodological work on how to properly model the transitional years between childhood and adulthood, as was done for BMI, is needed [38]. The visible mismatch between our paediatric curves and Prado's is expected based on the existing literature. Body composition, especially fat‐ and muscle‐mass, undergoes substantial and non‐linear changes around 17–19 years of age. Importantly, this mismatch is not unique to body composition and can also be observed in the transition from BMIz to adult BMI classification, where notable misclassification occurs especially around ages 17–19 years [38, 39]. We exercised minimal exclusion criteria in developing the reference curves. Youth with medical conditions, genetic disorders, or taking medications that may affect their growth, or their metabolic data could not be precisely identified given NHANES data. Whether these reference curves developed in the general population are useful in these subgroups, or whether they need to be modified, should be further investigated.

## 5 Conclusion

To the best of our knowledge, this is the first study to utilise the LMS method to develop ASMI and FMI reference curves and phenotypes for children aged 8–17 years old. These reference curves and phenotypes possess future clinical significance as they can potentially be used as a reliable tool to assess and diagnose clinical obesity in children, especially sarcopenic obesity. For instance, people with sarcopenic obesity (characterised by excessive fat mass in the presence of reduced muscle mass) have higher rates of illness, disability, and mortality compared to people with either low muscle mass or obesity [5] but are oftentimes misclassified by BMI. Indeed, more precise methods have been recently declared as necessary to confirm adiposity for a clinical obesity diagnosis [6]. Nevertheless, identification of ASMI and FMI severity would likely better represent health risks than a median split adjusted for age and sex. Further improvements to the reference curves and phenotype classification are needed.

## Author Contributions

S.T.S. and L.K. conceptualised and designed the study and interpreted the results. S.T.S. wrote the first version of the manuscript and conducted all analyses. C.F. coordinated revisions with L.K. and input from all other co‐authors. All authors approved the final version of the manuscript. M.H. is the principal investigator of QUALITY and guarantor of this work. M.H. and L.K. have full access to all the data in the study. L.K. takes responsibility for the integrity of the data and the accuracy of the data analysis.

## Ethics Statement

The study was conducted ethically in accordance with the Tri Council policy statement on the ethical conduct for research involving humans. Ethics review boards of Centre Hospitalier Universitaire Sainte‐Justine and the Quebec Heart and Lung Institute approved the study protocol (#MP‐21‐2005‐79, 2040). The secondary data analysis for this project was approved by the Concordia University ethics board (#0000840540).

## Consent

All parents provided informed consent, and study participants provided assent.

## Conflicts of Interest

The authors declare no conflicts of interest.

## Supporting information


**Table S1:** Appendicular skeletal mass index (kg/m^2^) L, M, S, and decile values for females.
**Table S2:** Fat mass index (kg/m^2^) L, M, S, and decile values for females.
**Table S3:** Appendicular skeletal mass index (kg/m^2^) L, M, S and decile values for males.
**Table S4:** Fat mass index (kg/m^2^) L, M, S, and decile values for males.

## Data Availability

The data that support the findings of this study are available on request from the corresponding author. The data are not publicly available due to privacy or ethical restrictions.
