# Supplementary material for: Development and Application of Children's Sex‐ and Age‐Specific Fat‐Mass and Muscle‐Mass Reference Curves From Dual‐Energy X‐Ray Absorptiometry Data for Predicting Cardiometabolic Risk
Source: Pediatr Obes. 2025 Aug 29;20(12):e70051. doi: 10.1111/ijpo.70051 (PMC12590095; doi:10.1111/ijpo.70051)
Supplement: Supplementary file 1 — Table S1: Appendicular skeletal mass index (kg/m2) L, M, S, and decile values for females. Table S2: Fat mass index (kg/m2) L, M, S, and decile values for females. Table S3: Appendicular skeletal mass index (kg/m2) L, M, S and decile values for males. Table S4: Fat mass index (kg/m2) L, M, S, and decile values for males. [file IJPO-20-e70051-s001.docx]

Table S1: Appendicular skeletal mass index (kg/m^2^) L, M, S, and decile values for females

|  |  |  |  | **Appendicular skeletal mass index (kg/m^2^) deciles** | | | | | | | | |
| --- | --- | --- | --- | --- | --- | --- | --- | --- | --- | --- | --- | --- |
| **Age**  **(years)** | **L** | **M** | **S** | **10^th^** | **20^th^** | **30^th^** | **40^th^** | **50^th^** | **60^th^** | **70^th^** | **80^th^** | **90^th^** |
| 8 | -1.460 | 4.793 | 0.155 | 4.026 | 4.254 | 4.439 | 4.614 | 4.793 | 4.991 | 5.226 | 5.540 | 6.060 |
| 9 | -0.694 | 5.159 | 0.154 | 4.287 | 4.556 | 4.769 | 4.964 | 5.159 | 5.368 | 5.607 | 5.911 | 6.382 |
| 10 | -0.511 | 5.489 | 0.154 | 4.548 | 4.841 | 5.071 | 5.281 | 5.489 | 5.709 | 5.961 | 6.276 | 6.758 |
| 11 | -0.880 | 5.751 | 0.154 | 4.794 | 5.087 | 5.320 | 5.535 | 5.751 | 5.984 | 6.254 | 6.599 | 7.143 |
| 12 | -1.082 | 5.962 | 0.154 | 4.985 | 5.281 | 5.518 | 5.739 | 5.962 | 6.205 | 6.488 | 6.856 | 7.445 |
| 13 | -1.066 | 6.109 | 0.154 | 5.106 | 5.410 | 5.653 | 5.880 | 6.109 | 6.358 | 6.648 | 7.026 | 7.628 |
| 14 | -0.900 | 6.197 | 0.155 | 5.163 | 5.479 | 5.730 | 5.963 | 6.197 | 6.450 | 6.742 | 7.118 | 7.711 |
| 15 | -0.842 | 6.236 | 0.156 | 5.184 | 5.506 | 5.762 | 5.998 | 6.236 | 6.492 | 6.788 | 7.167 | 7.762 |
| 16 | -1.160 | 6.228 | 0.158 | 5.193 | 5.504 | 5.755 | 5.989 | 6.228 | 6.488 | 6.794 | 7.195 | 7.843 |
| 17 | -1.737 | 6.194 | 0.160 | 5.199 | 5.490 | 5.728 | 5.957 | 6.194 | 6.460 | 6.781 | 7.219 | 7.977 |
| 18 | -0.733 | 6.487 | 0.172 | 5.302 | 5.664 | 5.952 | 6.219 | 6.487 | 6.776 | 7.111 | 7.540 | 8.214 |
| 19 | -0.728 | 6.508 | 0.173 | 5.316 | 5.680 | 5.970 | 6.238 | 6.508 | 6.799 | 7.136 | 7.568 | 8.247 |
| Note: Values for age 18 and 19 are obtained from Prado et al.’s (2014) results. | | | | | | | | | | | | |

Table S2: Fat mass index (kg/m^2^) L, M, S, and decile values for females

|  |  |  |  | **Fat mass index (kg/m^2^) deciles** | | | | | | | | |
| --- | --- | --- | --- | --- | --- | --- | --- | --- | --- | --- | --- | --- |
| **Age (years)** | **L** | **M** | **S** | **10^th^** | **20^th^** | **30^th^** | **40^th^** | **50^th^** | **60^th^** | **70^th^** | **80^th^** | **90^th^** |
| 8 | -0.529 | 5.322 | 0.385 | 3.432 | 3.945 | 4.392 | 4.838 | 5.322 | 5.883 | 6.589 | 7.595 | 9.432 |
| 9 | -0.444 | 5.759 | 0.391 | 3.663 | 4.236 | 4.733 | 5.227 | 5.759 | 6.373 | 7.141 | 8.220 | 10.152 |
| 10 | -0.379 | 6.092 | 0.396 | 3.829 | 4.450 | 4.988 | 5.520 | 6.092 | 6.748 | 7.563 | 8.700 | 10.704 |
| 11 | -0.344 | 6.289 | 0.398 | 3.929 | 4.579 | 5.140 | 5.695 | 6.289 | 6.969 | 7.811 | 8.979 | 11.024 |
| 12 | -0.337 | 6.495 | 0.397 | 4.059 | 4.731 | 5.310 | 5.883 | 6.495 | 7.195 | 8.061 | 9.261 | 11.355 |
| 13 | -0.357 | 6.760 | 0.393 | 4.254 | 4.945 | 5.541 | 6.130 | 6.760 | 7.481 | 8.373 | 9.611 | 11.777 |
| 14 | -0.401 | 6.966 | 0.386 | 4.438 | 5.134 | 5.735 | 6.329 | 6.966 | 7.696 | 8.601 | 9.861 | 12.081 |
| 15 | -0.464 | 7.233 | 0.376 | 4.684 | 5.385 | 5.990 | 6.590 | 7.233 | 7.973 | 8.894 | 10.183 | 12.474 |
| 16 | -0.542 | 7.482 | 0.365 | 4.932 | 5.632 | 6.237 | 6.837 | 7.482 | 8.226 | 9.156 | 10.467 | 12.825 |
| 17 | -0.625 | 7.618 | 0.354 | 5.111 | 5.798 | 6.392 | 6.982 | 7.618 | 8.354 | 9.278 | 10.590 | 12.981 |
| 18 | -0.196 | 8.658 | 0.419 | 5.195 | 6.155 | 6.981 | 7.794 | 8.658 | 9.639 | 10.840 | 12.480 | 15.275 |
| 19 | -0.185 | 8.760 | 0.419 | 5.253 | 6.227 | 7.064 | 7.887 | 8.760 | 9.751 | 10.961 | 12.611 | 15.414 |
| Note: Values for age 18 and 19 are obtained from Prado et al.’s (2014) results. | | | | | | | | | | | | |

Table S3: Appendicular skeletal mass index (kg/m^2^) L, M, S, and decile values for males

|  |  |  |  | **Appendicular skeletal mass index (kg/m^2^) deciles** | | | | | | | | |
| --- | --- | --- | --- | --- | --- | --- | --- | --- | --- | --- | --- | --- |
| **Age (years)** | **L** | **M** | **S** | **10^th^** | **20^th^** | **30^th^** | **40^th^** | **50^th^** | **60^th^** | **70^th^** | **80^th^** | **90^th^** |
| 8 | 0.297 | 5.337 | 0.136 | 4.462 | 4.750 | 4.966 | 5.156 | 5.337 | 5.524 | 5.728 | 5.975 | 6.328 |
| 9 | -0.748 | 5.458 | 0.133 | 4.652 | 4.904 | 5.101 | 5.280 | 5.458 | 5.647 | 5.862 | 6.133 | 6.546 |
| 10 | -1.109 | 5.678 | 0.130 | 4.873 | 5.121 | 5.317 | 5.497 | 5.678 | 5.872 | 6.095 | 6.380 | 6.827 |
| 11 | -0.902 | 5.989 | 0.136 | 5.093 | 5.370 | 5.589 | 5.789 | 5.989 | 6.203 | 6.448 | 6.759 | 7.241 |
| 12 | -0.603 | 6.361 | 0.150 | 5.304 | 5.633 | 5.891 | 6.127 | 6.361 | 6.611 | 6.895 | 7.254 | 7.804 |
| 13 | -0.396 | 6.847 | 0.173 | 5.538 | 5.944 | 6.264 | 6.556 | 6.847 | 7.156 | 7.509 | 7.953 | 8.633 |
| 14 | -0.558 | 7.340 | 0.158 | 6.056 | 6.455 | 6.768 | 7.055 | 7.340 | 7.644 | 7.991 | 8.430 | 9.104 |
| 15 | -0.891 | 7.729 | 0.144 | 6.514 | 6.888 | 7.184 | 7.456 | 7.729 | 8.021 | 8.357 | 8.786 | 9.456 |
| 16 | -1.028 | 7.979 | 0.148 | 6.711 | 7.098 | 7.406 | 7.691 | 7.979 | 8.290 | 8.650 | 9.115 | 9.850 |
| 17 | -1.093 | 8.130 | 0.146 | 6.860 | 7.246 | 7.555 | 7.841 | 8.130 | 8.442 | 8.804 | 9.273 | 10.016 |
| 18 | 0.288 | 8.329 | 0.146 | 6.888 | 7.361 | 7.716 | 8.029 | 8.329 | 8.637 | 8.976 | 9.384 | 9.971 |
| 19 | 0.314 | 8.360 | 0.146 | 6.918 | 7.391 | 7.746 | 8.059 | 8.360 | 8.668 | 9.007 | 9.416 | 10.003 |
| Note: Values for age 18 and 19 are obtained from Prado et al.’s (2014) results. | | | | | | | | | | | | |

Table S4: Fat mass index (kg/m^2^) L, M, S, and decile values for males

|  |  |  |  | **Fat mass index (kg/m^2^) deciles** | | | | | | | | |
| --- | --- | --- | --- | --- | --- | --- | --- | --- | --- | --- | --- | --- |
| **Age (years)** | **L** | **M** | **S** | **10^th^** | **20^th^** | **30^th^** | **40^th^** | **50^th^** | **60^th^** | **70^th^** | **80^th^** | **90^th^** |
| 8 | -0.789 | 4.539 | 0.382 | 3.001 | 3.408 | 3.769 | 4.135 | 4.539 | 5.020 | 5.645 | 6.576 | 8.426 |
| 9 | -0.682 | 4.748 | 0.400 | 3.058 | 3.506 | 3.902 | 4.305 | 4.748 | 5.275 | 5.956 | 6.964 | 8.930 |
| 10 | -0.598 | 4.978 | 0.419 | 3.126 | 3.616 | 4.050 | 4.492 | 4.978 | 5.555 | 6.299 | 7.394 | 9.506 |
| 11 | -0.556 | 5.097 | 0.434 | 3.139 | 3.654 | 4.113 | 4.581 | 5.097 | 5.709 | 6.501 | 7.668 | 9.917 |
| 12 | -0.558 | 5.066 | 0.444 | 3.090 | 3.608 | 4.070 | 4.543 | 5.066 | 5.691 | 6.502 | 7.705 | 10.049 |
| 13 | -0.597 | 4.961 | 0.447 | 3.032 | 3.534 | 3.984 | 4.446 | 4.961 | 5.578 | 6.385 | 7.594 | 9.996 |
| 14 | -0.643 | 4.786 | 0.445 | 2.943 | 3.421 | 3.850 | 4.292 | 4.786 | 5.381 | 6.164 | 7.349 | 9.747 |
| 15 | -0.667 | 4.679 | 0.444 | 2.889 | 3.352 | 3.769 | 4.198 | 4.679 | 5.259 | 6.026 | 7.191 | 9.571 |
| 16 | -0.669 | 4.760 | 0.442 | 2.944 | 3.414 | 3.837 | 4.273 | 4.760 | 5.348 | 6.125 | 7.303 | 9.707 |
| 17 | -0.657 | 4.815 | 0.439 | 2.984 | 3.459 | 3.886 | 4.325 | 4.815 | 5.404 | 6.179 | 7.349 | 9.713 |
| 18 | -0.0003 | 5.531 | 0.464 | 3.053 | 3.744 | 4.337 | 4.918 | 5.531 | 6.221 | 7.054 | 8.172 | 10.022 |
| 19 | 0.008 | 5.646 | 0.457 | 3.137 | 3.839 | 4.441 | 5.028 | 5.646 | 6.339 | 7.174 | 8.292 | 10.133 |
| Note: Values for age 18 and 19 are obtained from Prado et al.’s (2014) results. | | | | | | | | | | | | |
